# Engaging with complexity to improve the health of indigenous people: a call for the use of systems thinking to tackle health inequity

**DOI:** 10.1186/s12939-017-0521-2

**Published:** 2017-02-21

**Authors:** Alison Hernández, Ana Lorena Ruano, Bruno Marchal, Miguel San Sebastián, Walter Flores

**Affiliations:** 1 Center for the Study of Equity and Governance in Health Systems (CEGSS), Guatemala, Guatemala; 2 Center for International Health, University of Bergen, Bergen, Norway; 3 Institute of Tropical Medicine (ITM), Antwerp, Belgium; 4 Department of Public Health and Clinical Medicine, Umeå University, Umeå, Sweden

**Keywords:** Indigenous people, Systems thinking, Health systems, Equity, Indigenous health, Guatemala, Accountability

## Abstract

The 400 million indigenous people worldwide represent a wealth of linguistic and cultural diversity, as well as traditional knowledge and sustainable practices that are invaluable resources for human development. However, indigenous people remain on the margins of society in high, middle and low-income countries, and they bear a disproportionate burden of poverty, disease, and mortality compared to the general population. These inequalities have persisted, and in some countries have even worsened, despite the overall improvements in health indicators in relation to the 15-year push to meet the Millennium Development Goals. As we enter the Sustainable Development Goals (SDGs) era, there is growing consensus that efforts to achieve Universal Health Coverage (UHC) and promote sustainable development should be guided by the moral imperative to improve equity. To achieve this, we need to move beyond the reductionist tendency to frame indigenous health as a problem of poor health indicators to be solved through targeted service delivery tactics and move towards holistic, integrated approaches that address the causes of inequalities both inside and outside the health sector. To meet the challenge of engaging with the conditions underlying inequalities and promoting transformational change, equity-oriented research and practice in the field of indigenous health requires: engaging power, context-adapted strategies to improve service delivery, and mobilizing networks of collective action. The application of systems thinking approaches offers a pathway for the evolution of equity-oriented research and practice in collaborative, politically informed and mutually enhancing efforts to understand and transform the systems that generate and reproduce inequities in indigenous health. These approaches hold the potential to strengthen practice through the development of more nuanced, context-sensitive strategies for redressing power imbalances, reshaping the service delivery environment and fostering the dynamics of collective action for political reform.

The 400 million indigenous people worldwide represent a wealth of linguistic and cultural diversity, as well as traditional knowledge and sustainable practices that are invaluable resources for human development [1]. However, indigenous people remain on the margins of society in high, middle and low-income countries, and they bear a disproportionate burden of poverty, disease, and mortality compared to the general population [2, 3]. These inequalities have persisted, and in some countries have even worsened, despite the overall improvements in health indicators in relation to the 15-year push to meet the Millennium Development Goals [4, 5]. Attention garnered by the social determinants of health framework has enriched the understanding we have of the complex conditions that give rise to inequalities in indigenous health. This lens has brought into focus the structural and socio-political factors that contribute to health inequalities, illuminating the intersecting conditions of poverty, social and political exclusion, discrimination and land loss that shape indigenous people’s health [6–8]. While the vital importance of addressing these conditions to improve indigenous health has become clear, the role of health equity-oriented research and practice in supporting systemic change requires urgent attention.

As we enter the Sustainable Development Goals (SDGs) era, there is growing consensus that efforts to achieve Universal Health Coverage (UHC) and promote sustainable development should be guided by the moral imperative to improve equity [9]. To achieve this, we need to move beyond the reductionist tendency to frame indigenous health as a problem of poor health indicators to be solved through targeted service delivery tactics and move towards holistic, integrated approaches that address the causes of inequalities both inside and outside the health sector [8]. The pressing question today is how equity-oriented research and practice can evolve to better engage with the complex conditions that underlie indigenous health inequalities, and promote processes of change that will transform the societies in which these inequalities are embedded.

The paradigm of systems thinking is well positioned to play a prominent role in re-orienting our perspectives and enabling researchers and practitioners to analyze and act upon the complex causes of inequalities in indigenous health. At its core, systems thinking is a way of thinking applied in approaching problems and designing solutions [10, 11]. This approach enables shifting from isolated, linear views of causes and outcomes to complex views of the patterns of interactions within the system of interest and emergent system behaviors. As an applied research paradigm, systems thinking offers tools and strategies that embrace complexity and build knowledge and skills to strengthen transformational processes [12]. The potential of these approaches to generate structural and human change as well as provide valuable information for decision-makers has been demonstrated in public health areas including tobacco control, emergency system performance, and health sector reforms [13, 14]. In the field of indigenous health, application of systems thinking approaches can enable a shift from a health-outcomes focus to seeing the whole system in which the well-being of indigenous populations is embedded, and integrating transformative action with a broader view of the interdependent elements, networks of relationships, and patterns of interaction that shape and impact health.

At the Center for the Study of Equity and Governance in Health Systems (CEGSS by its initials in Spanish) in Guatemala, our work during the past decade has focused on engaging with the governance structures and conditions of marginalization that impact indigenous health. Indigenous people of 23 ethnicities make up 45% of Guatemala’s 14 million inhabitants. They experience stark inequalities in income, health and education resulting from decades of social and economic exploitation, and were particularly targeted by the country’s military dictatorships during the 36 year-long internal war. This conflict left 200,000 victims, who were predominantly indigenous, and contributed to the worsening of already weak public services in rural and impoverished areas [15]. In our primary line of work, CEGSS supports the development of a citizen-led initiative for state accountability for the right to health in rural indigenous municipalities. Through our experiences, we have gained critical insights into the complex nature of pathways to redressing inequity and the potential of systems thinking approaches to enhance efforts to promote indigenous health. To meet the challenge of engaging with the conditions underlying inequalities and promoting transformational change, equity-oriented research and practice in the field of indigenous health requires: engaging power, context-adapted strategies to improve service delivery, and mobilizing networks of collective action. In the following sections, we elaborate on what these paths of action have meant in our work with indigenous people in Guatemala, and the relevance of systems thinking approaches for catalyzing their contribution to equity in indigenous health.

## Engaging power

For indigenous people, health inequity is an expression of inequity of power. Governance is a critical point of focus in efforts to redress imbalances in a country’s economic, social and political institutions [16]. Its quality is determined by the degree to which its institutions and processes are transparent and accountable to the public, and people are able to participate in decisions that affect their lives [17]. Guatemala, like many other Latin American countries, has progressive legislation in place that establishes a framework for social participation through a structured scheme of citizen councils from the community to the national level. In practice, however, indigenous citizens’ capacity to participate and advocate for their interests and rights in these decision-making forums is limited by historical processes and current institutional practices of social exclusion. Tackling these deep-rooted inequities requires facilitating the political and legal empowerment of indigenous people. Concretely, this work has involved human rights literacy and capacity building in public policies and the legal framework for participation, as well as training in skills for negotiation and advocacy with state authorities and other public entities that oversee legal compliance and human rights protection. While shifting power imbalances is a long-term endeavor, indigenous leaders have been persistent in submitting formal demands for resolution of health system deficiencies at the municipal, provincial and national levels, and in some cases have established regular participation in municipal councils and dialogue spaces with provincial health authorities.

Engaging power implies engaging with the complexity of transforming the social, political, cultural, ecological, and economic systems that shape indigenous health. Efforts to facilitate the empowerment of indigenous people so they can become agents of change in these systems can contribute to the strengthening of their voice in the governance environment and enhance the state’s accountability and responsiveness to their needs. Systems thinking approaches can support these aims by deepening understanding of the interrelationships that underlie public sector function as well as the “soft” systems of formal and informal rules, norms and values that guide stakeholder action. Systems thinking tools, such as causal loop diagrams, behavior over time diagrams, and concept mapping are compatible with complex-sensitive approaches to governance, and can be used to engage citizen and state stakeholders in joint analysis, integrate different perspectives and elicit the mental models that are operating [12, 13].

## Context-adapted strategies to improve service delivery

Improving service delivery to ensure equitable universal coverage requires strategic efforts to address the myriad factors limiting indigenous people’s access to health care. Discrimination, abusive treatment, language barriers, inadequate infrastructure, medicines and equipment, and the exclusionary biomedical model of care are among the main barriers they face in seeking services [18–20]. However, the “best practice” interventions to improve specific health indicators that are rolled out and scaled up in global health initiatives do little to account for the cultural and structural contexts of service delivery that determine their capacity to reach the needs of indigenous people [21]. A shift to context-adapted strategies implies developing multi-level action processes to reshape the conditions that limit the accessibility of care. Indigenous citizen-led efforts in Guatemala have focused on monitoring services to gather user complaints and audio-visual evidence of the problems they experience, raising awareness about the right to health and mechanisms for reporting rights violations, and establishing communication channels and collaboration with district health authorities to seek solutions. Civil society initiatives in Guatemala and other Latin American countries have led to the development of intercultural models of primary health care that integrate local indigenous and western medicine, and in several countries intercultural health policies have been adopted to mandate the incorporation of these models in the public health services [22–24]. These types of approaches are inherently complex as they entail on-going interaction between multiple stakeholders with different, and sometimes opposing, agendas in order to achieve and maintain improvements in the service delivery environment [25]. Dialogue and relationship building between health system actors and indigenous service users are central to addressing tensions, changing mindsets and the emergence of practices of respectful and culturally appropriate care.

A systems-thinking lens is needed to avoid an isolated view of the effectiveness of particular interventions to improve health and gain understanding of the complex pathways of strategic approaches to redress barriers to access. Optimal implementation of context-adapted strategies requires inputs from research that sheds light on emergent processes and the underlying social mechanisms of equity-oriented change in service delivery. Complexity-sensitive evaluation approaches, such as realist evaluation, can be useful for testing the program logic or theory behind strategic multi-level action processes, and can be a valuable tool to assess their impact in a way that sheds light on what works, for whom, and under what conditions [26]. These approaches typically combine quantitative and qualitative tools to identify the underlying mechanisms that explain “how” the outcomes were caused and the influence of context. Implementation of context-adapted approaches should also be supported by monitoring and evaluation systems that capture heterogeneity in program strategies and emerging processes across sites, and connect to feedback loops and participatory learning mechanisms. Through such tools, strategic approaches can be underpinned by continuous learning that enables adaptation in response to changing conditions, windows of opportunity and new insights into mechanisms of change [17].

## Mobilizing networks of collective action

The social, economic and political forces that have caused the historical exclusion of indigenous people are enormous. Efforts to reverse these conditions and generate structural changes that promote equity in the health system and beyond are typically met by resistance from powerful elites who seek to protect their interests. Citizen-led actions to demand state accountability for indigenous people’s right to health through higher allocation of public resources and fair and just procedures to resolve grievances require networks of support to connect local actions to broader social forces of national scope. Mobilization of networks of collective action implies forming strategic alliances with other pro-reform associations and applying diverse tactics to generate political pressure [27]. In Guatemala, this has meant linking up the municipal-level associations of indigenous leaders to jointly communicate their demands for structural change in national forums, as well as developing a civil society coalition to plan actions to influence public opinion and political processes to support universal coverage and the right to health for all Guatemalans. This kind of network-building efforts contributes to amplify the voices of indigenous citizens and also strengthens the position of national level coalitions by connecting them to a grassroots base of citizen-generated evidence and demands [28].

Achieving equity in indigenous health requires engaging in the highly interactive and dynamic political process of generating structural changes in health system financing and resource allocation [29]. To increase the likelihood that such policy reforms will reach the national agenda and be supported, we need to understand key stakeholder incentives as well as the structural and relational qualities of the networks of indigenous citizens and organizations that implement coordinated actions to drive change. Social network analysis is a useful tool that can be used to improve understanding of the evolving networks of communication, collaboration and conflict that connect the actors and organizations engaged in advocacy action with each other and with state actors. This knowledge can guide politically-informed, vertical and horizontal integration of strategies that would expand the scale and impact of local and national efforts to demand pro-equity structural reforms [30].

## Conclusion

The application of systems thinking approaches offers a pathway for the evolution of equity-oriented research and practice in collaborative, politically informed and mutually enhancing efforts to understand and transform the systems that generate and reproduce inequities in indigenous health. These approaches hold the potential to strengthen practice through the development of more nuanced, context-sensitive strategies for redressing power imbalances, reshaping the service delivery environment and fostering the dynamics of collective action for political reform. To harness the transformative potential of systems thinking, its tools and strategies should be applied through partnerships that engage researchers with the actors on the front-lines of change – community-based and civil society organizations, health care providers and managers, and policy makers. This requires new capacities for combining scholarly, operational and political work in collaboratively defined research agendas that directly engage with processes to promote equity for indigenous people.
